# Supplementary material for: Alterations in Th17 Cells and Non-Classical Monocytes as a Signature of Subclinical Coronary Artery Atherosclerosis during ART-Treated HIV-1 Infection
Source: Cells. 2024 Jan 15;13(2):157. doi: 10.3390/cells13020157 (PMC10813976; doi:10.3390/cells13020157)
Supplement: Supplementary file 1 [file cells-13-00157-s001.zip › TRWS Suppl Files 2024-01-04/TRWS et al. HIV CVD Suppl Table 1 2024-01-04.pdf]

Supplemental Table 1: Antibodies and dyes used for polychromatic flow cytometry

Target	Clone	Fluorochrome	Supplier
**Th17/Treg cocktail**			
CD3	UCHT1	Alexa Fluor 700	Biolegend
CD4	RPA-T4	PerCP/Cyanine5.5	Biolegend
CD45RA	HI100	APC-eFluor 780	eBioscience
CCR6	11A9	PE	BD Pharmingen
CCR7	3D12	BV786	BD Horizon
CD25	M-A251	BV421	BD Horizon
CD127	HIL-7R-M21	PE-Cy7	BD Pharmingen
CD26	M-A261	APC	BD Pharmingen™
CD161	DX12	BV650	BD Horizon
FOXP3	PCH101	Alexa Fluor 488	eBioscience
LIVE/DEAD™ Fixable Aqua Dead Cell Stain			Thermo Fisher Scientific
**Monocyte cocktail**			
CD3	UCHT1	Alexa Fluor 700	Biolegend
CD4	RPA-T4	PerCP/Cyanine5.5	Biolegend
CD1c	F10/21A3	BV786	BD OptiBuild
CD14	M5E2	APC	BD Pharmingen
CD16	3G8	PE-Cy7	BD Pharmingen
HLA-DR	L243	APC/Cyanine7	Biolegend
CCR6	11A9	PE	BD Pharmingen
M-DC8/Slan	DD-1	FITC	Miltenyi
CCR2	K036C2	PE/Dazzle 594	Biolegend
CX3CR1	2A9-1	BV421	BD Horizon
LIVE/DEAD™ Fixable Aqua Dead Cell Stain			Thermo Fisher Scientific
**Monocyte and DC cocktail**			
CD3	UCHT1	Alexa Fluor 700	Biolegend
CD4	RPA-T4	PerCP/Cyanine5.5	Biolegend
CD1c	F10/21A3	BV786	BD OptiBuild
CD14	M5E2	APC	BD Pharmingen
CD16	3G8	PE-Cy7	BD Pharmingen
HLA-DR	L243	APC/Cyanine7	Biolegend
CCR6	11A9	BV421	BD Horizon
CCR9	112509	PE	BD Pharmingen
CD123	6H6	PE/Dazzle 594	eBioscience
CD303 (BDCA2)	AC144	FITC	Miltenyi
LIVE/DEAD™ Fixable Aqua Dead Cell Stain			Thermo Fisher Scientific
